# Citral Induced Apoptosis through Modulation of Key Genes Involved in Fatty Acid Biosynthesis in Human Prostate Cancer Cells: *In Silico* and *In Vitro* Study

**DOI:** 10.1155/2020/6040727

**Published:** 2020-03-18

**Authors:** Sri Renukadevi Balusamy, Haribalan Perumalsamy, Karpagam Veerappan, Md. Amdadul Huq, S. Rajeshkumar, T. Lakshmi, Yeon Ju Kim

**Affiliations:** ^1^Department of Food Science and Biotechnology, Sejong University, Gwangjin-gu, Seoul, Republic of Korea; ^2^Graduate School of Biotechnology, College of Life Science, Kyung Hee University, Yongin 446-701, Republic of Korea; ^3^Department of Food Nutrition, Chung Ang University, Anseong-si, Gyeonggi-do 17546, Republic of Korea; ^4^Department of Pharmacology, Saveetha Dental College and Hospitals, Saveetha University, SIMATS, Chennai 600077, TN, India

## Abstract

The isomers of citral (*cis*-citral and *trans*-citral) were isolated from the *Cymbopogon citratus* (DC.) Stapf oil demonstrates many therapeutic properties including anticancer properties. However, the effects of citral on suppressing human prostate cancer and its underlying molecular mechanism have yet to be elucidated. The citral was isolated from lemongrass oil using various spectroscopic analyses, such as electron ionized mass spectrometry (EI-MS) and nuclear magnetic resonance (NMR) spectroscopy respectively. We carried out 3-(4,5-dimethylthiazol-2-yl)-2,5-diphenyl tetrazolium bromide (MTT) assay to evaluate the cell viability of citral in prostate cancer cells (PC-3 and PC3M). Furthermore, to confirm that PC3 undergoes apoptosis by inhibiting lipogenesis, we used several detection methods including flow cytometry, DNA fragmentation, Hoechst staining, PI staining, oil staining, qPCR, and Western blotting. Citral impaired the clonogenic property of the cancer cells and altered the morphology of cancer cells. Molecular interaction studies and the PASS biological program predicted that citral isomers tend to interact with proteins involved in lipogenesis and the apoptosis pathway. Furthermore, citral suppressed lipogenesis of prostate cancer cells through the activation of AMPK phosphorylation and downregulation of fatty acid synthase (FASN), acetyl coA carboxylase (ACC), 3-hydroxy-3-methylglutaryl-coenzyme A reductase (HMGR), and sterol regulatory element-binding protein (SREBP1) and apoptosis of PC3 cells by upregulating *BAX* and downregulating *Bcl-2* expression. In addition, i*n silico* studies such as ADMET predicted that citral can be used as a safe potent drug for the treatment of prostate cancer. Our results indicate that citral may serve as a potential candidate against human prostate cancer and warrants *in vivo* studies.

## 1. Introduction

Prostate cancer is the second most leading cancer in Western countries [1]. However, the prevalence of prostate cancer in Asian countries are lower and considered to be the fifth most common cancer among Korean men population [2]. In recent years, the incidence of prostate cancer in Korea is rapidly increasing. According to the Korean National Cancer Incidence Database, the age-standardized incidence report suggests that the annual percent change in prostate cancer was 11.4% which is the second-largest cancer observed following the thyroid cancer [3]. A decrease in the mortality rate of prostate cancer patients may be attributed to early diagnosis. Current therapeutic measures fail to cure the malignancy and life span can be extended only for 4–6 months denoting that chemoprevention of prostate cancer is the main approach to reduce the morbidity [4]. Therefore, identifying the novel drug from natural products can be the most effective and alternative therapy to reduce the mortality of prostate cancer. Thus, the researchers are highly focusing on natural products for the prevention of many cancers.

There is an increasing evidence that metabolic reprogramming plays a significant role in the development of cancer and disease progression [5]. An increase in fatty acid metabolism is linked to altered cancer cell metabolism. There are several studies conducted to prove the link between fatty acid synthesis and cancer progression including prostate cancer [6], pancreatic cancer [7], hepatocellular carcinoma [8], and breast cancer [9]. Therefore, identifying the target that inhibits the genes and enzymes involved in fatty acid synthesis can reduce the growth of the tumor cells and increase the life span of a cancer patient.

*Cymbopogon citratus* (DC.) Stapf. commonly known as lemongrass is extensively used as a medicinal plant in folk medicine for the treatment of various diseases as it has antimutagenic, antiproliferative, and antiparasitic properties. By the process of steam distillation, a volatile oil is obtained from the leaves of lemongrass. The pharmacological properties of lemongrass were due to the presence of citral which is an acyclic monoterpene. Many studies reported that lemongrass oil possesses many pharmacological properties such as antimicrobial [10] and insecticidal [11]properties; only few studies demonstrated the anticancer properties of lemongrass, for instance, cervical cancer, HeLa and ME-180 cells [12], breast cancer (MCF-7) cells [13], prostate cancer, PC3, and LNCap [14]. However, to date its molecular mechanism in prostate cancer cells has not been elucidated. Our present study isolated citral from *C. citratus *combined both *in silico* and *in vitro* analyses to reveal the potential antiproliferative activity of citral as a possible candidate to induce apoptosis by targeting lipogenesis pathway.

## 2. Materials and Methods

### 2.1. Instrumental Analysis

The ^1^H and ^13^C NMR spectra were recorded in DMSO on an AVANCE 600 spectrometer (Bruker, Rheinstetten, Germany) at 600 and 150 MHz, respectively, using tetramethylsilane as an internal standard. The chemical shifts are given in *δ* (ppm). The DEPT spectra were acquired using the Bruker software. The UV spectra were obtained in ethanol or methanol on a UVICON 933/934 spectrophotometer (Kontron, Milan, Italy) and the mass spectra on a JMS-DX 303 spectrometer (Jeol, Tokyo, Japan). Silica gel 60 (0.063–0.2 mm) (Merck, Darmstadt, Germany) was used for column chromatography. Merck precoated silica gel plates (Kieselgel 60F 254) were used for analytical thin-layer chromatography (TLC). An Isolera one medium-pressure liquid chromatograph (Biotage, Uppsala, Sweden) and an Agilent 1200 high-performance liquid chromatograph (Agilent, Santa Clara, CA, USA) were used to isolate the active compounds.

### 2.2. Materials

Commercially available anticancer agent cisplatin and 3-(4,5-dimethylthiazol-2-yl)-2,5-diphenyl tetrazolium bromide (MTT) were purchased from Sigma-Aldrich (St. Louis, MO). Rosewell Park Memorial Institute (RPMI) 1640 medium, Dulbecco's Modified Eagle's Medium (DMEM), and Fetal Bovine Serum (FBS) were supplied by Life Technologies (Grand Island, NY). Phosphate-buffered saline (PBS) was purchased from Sigma-Aldrich. Antibiotic-antimycotic solution and 0.5% trypsin-ethylenediaminetetraacetic acid (EDTA) were purchased from Invitrogen (Grand Island, NY, USA). Maxima SYBR Green/ROX qRT-PCR Master Mix was supplied by Thermo Scientific (Foster, CA, USA). All the other chemicals and reagents used in this study were reagent-grade quality and available commercially.

### 2.3. Antibodies

The primary antibodies used in this study were anti-β actin antibody (sc-47778), anti-BAX rabbit polyclonal antibody (sc-493), Bcl-2 rabbit polyclonal antibody (sc-492), AMPKα1 (sc-398861), ACC (sc-137104), fatty acid synthase antibody (sc-55580), SREBP1 (sc-13551), and HMGCR (sc-271595), and the secondary antibodies used in this study were goat anti-rabbit immunoglobulin G (IgG) H & amp, L (HRP) (sc-2054), goat anti-mouse IgG-HRP (sc-2005), and mouse anti-rabbit IgG-HRP (sc-2357). All the above antibodies were purchased from Santa Cruz Biotechnology (Dallas, TX, USA).

### 2.4. Medium-Pressure Liquid Chromatography (MPLC)

The lemongrass oil (5 g) was monitored by TLC on silica gel plates (Silica gel 60 F_254_) developed with hexane and ethyl acetate solvent system as previously described [15]. Based on TLC pattern, it was separated by MPLC using a Biotage Isolera apparatus equipped with a UV detector at 254 nm and 365 nm and a column cartridge SNAP (100 g silica gel) with column volume 132 mL. Separation was achieved with a gradient of hexane and ethyl acetate (100 : 0 (264 mL), 9 : 1 (396 mL), 8 : 2 (396 mL), 7 : 3 (660 mL), 6 : 4 (264 mL), 5 : 5 (264 mL), 3 : 7 (264 mL), and 1 : 9 (132 mL), by volume) at a flow rate 25 mL/min to provide 105 fractions (each about 22 mL). Column fractions were monitored by TLC on silica gel plates (Silica gel 60 F_254_) developed with hexane and ethyl acetate (8 : 2 by volume) mobile phase. Fractions with similar *R*_f_ values on the TLC plates were pooled. Spots were detected by spraying with 5% sulfuric acid and then heating on a hot plate as stated previously. A preparative high-performance liquid chromatography (HPLC) was performed to separate the constituents from active fractions. The column was a 7.8 mm i.d. × 300 mm *µ*Bondapak C18 (Waters, Milford, MA, USA) with a mobile phase of methanol and water (85 : 15 by volume) at a flow rate of 1 mL·min^–1^. Chromatographic separation was monitored using a UV detector at 233 nm. Finally, an active constituent 1 (citral) (28 mg) was isolated at a retention time of 13.8 min.

### 2.5. Prediction of Pharmacokinetic and Biological Activity

The pharmacokinetic (ADMET: absorption, distribution, metabolism, excretion, and toxicity) and drug-likeness (Lipinski rule of five) properties were calculated for neral and geranial molecules using SwissADME [16] and PreADMET web servers [17]. The ADMET properties such as human intestinal absorption (HIA), plasma protein binding ability (PPB), blood-brain barrier (BBB), and toxicity were predicted for *cis*-neral and *trans*-geraniol drug efficiencies. In addition, drug-likeness activities were predicted based on Lipinski's rule. Further, prediction of activity spectra for substances (PASS) web server was used for predicting putative biological activity [18] based on *cis*-neral and *trans*-gereniol chemical structures. This program resulted in a list of putative biological activities with a chance of probability scores [19]. The pharmacokinetic properties, drug-likeness, and biological activity were calculated based on SMILES (simplified molecular-input line-entry system) of chemical structures.

### 2.6. Molecular Interaction Studies

The three-dimensional crystal structures of B-cell lymphoma 2 (Bcl-2) and fatty acid synthase (FASN) were used for docking simulations with neral and geranial molecules. Bcl-2 (PDB ID: 4LVT) [20] with navitoclax and FASN (PDB ID: 2PX6) with orlistat [21] were downloaded from RCSB protein databank. For docking simulations, atomic coordinates were separated from complex structure, hydrogen atoms were added, and all water molecules were removed. Further, known inhibitor molecules were used as positive controls and their interaction sites were considered as the most favorable regions for docking reproducibility [22]. In case of ligands, their chemical structure was drawn using ChemSketch program and later was converted into a three-dimensional (3D) structure by using Discovery Studio Visualizer (DS, V17.2.0) (DS, http://www.accelrys.com; Accelrys, Inc. San Diego, CA, USA). The AutoDock Vina [23] program was implemented to find out the molecular level interactions, and the results were visualized by using DS. The detailed protein, ligand preparation, and docking parameters were described previously [24].

### 2.7. Cell Lines and Culture Conditions

Two human prostate cancer cell lines used in this study were as follows: PC3, human prostate cancer cell line; and PC3M, metastatic-derived variant of human prostate cancer cell line; they were purchased from the American Type Culture Collection (ATCC) (Manassas, VA, USA). One human lung normal cell MRC-5 (human fetal lung fibroblast cell line) was purchased from the Korean Cell Line Bank (Seoul, South Korea). All cell lines were cultured with RPMI 1640 or DMEM containing 10% FBS and 1% antibiotic-antimycotic solution under 5% CO_2_ at 37°C.

### 2.8. Cell Viability Assay

The cell cytotoxicity of citral toward the human prostate cancer cells (PC3 and PC-3M) was evaluated using an MTT assay performed as described previously [25, 26]. 10x MTT stock solution (5 mg/mL) was dissolved in phosphate-buffered saline (PBS) (pH 7.4), filter sterilized, and stored at −20°C. We used 2 × 10^4^ cells per well (96-well plates) containing 100 *μ*l of the complete culture medium. After 24 h, the citral test samples of different concentrations were added onto 96-well plates. The final concentration of DMSO Hybri-Max in all assays was less than 0.1%. Based on the preliminary test results, the antiproliferative activity of citral for PC3 was treated with 0, 5, 10, 15, 25, 50, 100 *μ*g/ml concentration whereas, for PC3M cells, citral was treated with 0, 3.75, 7.5, 12.5, 25, 50, 100 *μ*g/ ml concentrations, respectively. The culture plates were incubated for 2 days at 37°C supplied with humidified atmosphere of 5% CO_2_. The plates were then washed with 100 *μ*l PBS and 0.05% of 100 *μ*l MTT reagent was added to each well and then incubated for 4h as stated previously. MTT solution was removed and 200 *μ*l DMSO was added to each well. Finally, the plate was shaken in a microplate shaker for 10 min in dark condition to dissolve the purple formazan crystals. Cisplatin served as a positive control and was similarly formulated. The DMSO solution is used as a negative control. The optical density values were recorded using a VersaMax microplate reader (Molecular Devices, Sunnyvale, CA, USA) at 560 nm and 670 nm, respectively. Blank values were subtracted from experimental values.

### 2.9. Light Microscopy

The PC3 and PC-3M cell lines were cultured under the same conditions as those used for the cell maintenance. Cells were seeded into 96-well culture plates at a density of 2 × 10^4^ cells per well and grown for overnight. The cells were treated with or without citral (0, 3.75, 7.5, 12.5, 25, 50, 100 *μ*g/ml) in 0.1% DMSO for 48 h. Cisplatin served as a reference control and was similarly prepared to that of citral. DMSO solution served as a negative solution. Two days after treatment, a morphological characteristic of treated and untreated cells was observed through a Leica DMIL LED equipped with an Integrated 5.0 Mega-Pixel MC 170 HD camera (Wetzlar, Germany).

### 2.10. Colony Forming Assay

1 × 10^3^ cells of PC-3 were seeded into 6-well sterile plates as described previously [25, 26]. After adhesion, the cells were treated with different concentrations of citral ranging between ,(0, 5, 10, 15, 25, 50, 100 *μ*g/ml) respectively. Control wells were treated with only 0.1% DMSO. A day after incubation, the media were aspirated, and fresh media were added and incubated for 7 days at 37°C incubator supplied with 5% CO_2_. The clones developed were fixed with glutaraldehyde, stained using crystal violet, counted, and graphically plotted. Experiments were performed in triplicate and statistical analyses were carried out using Student's *t*-test.

### 2.11. AnnexinV-FITC/PI Staining Assay

AnnexinV-FITC/PI staining was performed as described previously [25, 26], by following the protocol of the AnnexinV-FITC Apoptosis Detection Kit (BD Pharmingen, San Diego, CA, USA). In brief, PC-3M cells (2 × 10^5^/well) were treated with different concentrations of citral (10 and 20 *μ*g/ml) for two days. Cells were harvested, washed twice with 1 mL cold PBS, and centrifuged at 300 × g for 5 min. Later, cells were suspended in 100 *µ*l of binding buffer containing 5 *µ*l AnnexinV-FITC and 5 *µ*l PI staining solution. Cells were incubated at room temperature for 15 min under dark condition. At last, 400 *µ*l of binding buffer was added before the analysis by BD Biosciences FACS Aria II flow cytometer (San Jose, CA, USA). The data were analyzed using BD Biosciences FACSDiva software 2.13.

### 2.12. Hoechst Staining

Citral induced apoptosis was performed as described previously [25, 26] by Hoechst staining 33342 kits according to the manufacturer's instructions with slight modification. At first, cells were washed twice with PBS and fixed with 4% paraformaldehyde for 10 min. Following that, cells were stained with (10 *μ*g/ml) Hoechst staining solution at 37°C for 10 min. The stained cells were washed 3 times with PBS, and imaging was captured under fluorescence microscope using a Leica DMLB fluorescence microscope (Wetzlar`, Germany).

### 2.13. Apoptotic Cell Propidium Iodide Staining

5 × 10^4^/well PC3 cells were seeded and apoptosis induction was measured as stated previously [26]. All wells except control wells were treated with (10 and 20 *μ*g/ml) of citral. The attached cells were then washed with 1 ml PBS and fixed with 4% paraformaldehyde about 15 minutes. Later, fixed cells were washed with 1 ml PBS and stained with 500 *μ*l propidium iodide (PI) (5 *μ*g/mL) solution at room temperature for 10 min. Apoptotic cells with condensed and fragmented nuclei were observed under fluorescence microscope using a Leica DMLB fluorescence microscope (Wetzlar, Germany).

### 2.14. Quantitative Real-Time Reverse Transcription-PCR Analysis

Treated and nontreated cultures of PC3 cell monolayers grown in 25 cm^2^ cell culture flasks (Corning Costar, NY, USA) were treated with (5, 10 and 20 *μ*g/ml) of citral. After 48 h of treatments, the total RNA was extracted from nontreated and treated cultures using RNeasy Mini Kit (Qiagen, Hilden, Germany). For real-time quantitative PCR (qRT-PCR), 500 ng of total RNA was reverse transcribed using oligo (dT)15 primer (0.2 mM) and AMV Reverse Transcriptase (10 U·*μ*l^−1^), and cDNA was synthesized using superscript First-Strand Synthesis Kit (Invitrogen, Carlsbad, CA) according to the manufacturer's instructions. qRT-PCR was performed in 96-well plate using 100 ng of cDNA in a 20 *μ*l reaction volume using SYBR® Green Sensimix Plus Master Mix (Quantace, Watford, England). Gene-specific primers used in this study were listed in Table 1. The melting point analysis of PCR products was carried out, which resulted in a single peak, indicating the presence of a single PCR product amplification. The thermal cycler conditions recommended by the manufacturer were used as follows: 10 min at 95°C, followed 40 cycles of 95°C for 10 s, 58°C for 10 s, and 72°C for 20 s. The fluorescent product was detected at the last step of each cycle and measured in the real-time reverse transcriptase PCR thermocycler, and its genomic increase of the fluorescence corresponding to the exponential increase of the product was used to determine the threshold cycle (Ct) in each reaction using the formula 2^−∆∆Ct^. The housekeeping gene encoding *β*-actin was used as a standard for all samples. All the real-time experiments were performed in triplicate, and the statistical analysis was determined using Student's *t*-test.

### 2.15. Western Blot Analysis

As described previously [25, 26], 2 × 10^5^/well PC3 prostate cancer cell line was treated with three concentrations (5, 10 and 20 *μ*g/ml) of citral in 0.1 mL DMSO for about two days. The cells were then harvested and washed twice with cold PBS. The cell pellets were lysed using RIPA lysis buffer (Sigma-Aldrich). The controls received 0.1% DMSO. The lysates were then centrifuged at 12,000 rpm for 20 min at 4°C. The protein content of the supernatant was determined using a Bradford Protein Assay kit, and BSA was used as the standard. The total proteins (15 *μ*g) were mixed with an equal volume of 5× sample buffer containing 40 mM of DL-dithiothreitol, boiled for 10 min and then loaded onto 10% sodium dodecyl sulfate-polyacrylamide gels using a Mini-Protean 3 electrophoresis cell (Bio-Rad, Hercules, CA, USA). After electrophoresis at 150V in 1.5 h, the proteins from the gels were transferred onto a polyvinyl difluoride membrane (Pall Corporation, Pensacola, FL, USA) using an electroblotting apparatus. The membrane was then blocked with 5% skim milk (BD Difco, Franklin Lakes, NJ, USA) in PBS containing 0.1% (v/v) Tween-20 (PBS-T) at room temperature for 1h and further incubated overnight at 4°C with a specific monoclonal or polyclonal antibody stated previously; each of them was used at a dilution of 1 : 1000 dilution. After washing with 0.1% PBS-T three times at 10-minute intervals, the membranes were further incubated with a goat anti-rabbit IgG H&L (HRP) secondary antibody at a 1 : 5000 dilution for 2 h RT. Finally, after washing with 0.1% PBS-T three times with a 10-minute interval between washes, the blots were developed with an ECL chemiluminescence reagent and immediately exposed to a CP-PU X-ray film (AGFA, Mortsel, Belgium). The differences in protein expression were quantified using a Molecular Imager Gel Doc XR system (Bio-Rad, Hercules, CA, USA) and normalized to actin expression on the same membrane.

### 2.16. DNA Fragmentation

2 × 10^5^/well PC3 prostate cancer cell line was treated with different concentrations of citral to determine DNA fragmentation. After 48 h of citral treatment, the cells were trypsinized and centrifuged at 1500 rpm for 5 min and supernatant was discarded. The pellet was washed twice with 1x PBS. Isolation of genomic DNA was carried out by Exgene cell SV (GeneAll Biotech, Korea) according to the manufacturer's instruction. DNA concentration was quantified using nanodrop, and equal volume of DNA (200 ng) was loaded with 6× loading buffer and run onto 0.8% agarose gel electrophoresis containing ethidium bromide in a Mini Gel Tank containing tris-borate-EDTA for 2 h at 80 V. The gel was then examined under ultraviolet light and photographed.

### 2.17. Oil Staining

The lipids in cancer cells were visualized using Oil Red O Staining. As described previously [25, 26], cells were washed with PBS and fixed in 4% paraformaldehyde for about 30 min. Later, the cells were stained using 0.5% Oil Red Staining Solution for 15 min in 60°C. Finally, the stained cells were washed thrice with 1 × PBS and were photographed under the light microscope.

### 2.18. Data Analysis

Antiproliferative activity was indicated as 50% inhibitory concentration (IC_50_) of the compound that decreased the cell viability up to 50% compared with untreated controls. The IC_50_ values were determined using GraphPad Prism 5 software program (GraphPad Software, La Jolla, CA). The IC_50_ values of all cancer cell lines and treatments were significantly different from one another when their 95% confidence limits (CLs) did not overlap. The percentage of growth inhibition was determined using the formula: % growth inhibition = (ODt/ODc) × 100, where ODt and ODc are the absorbance values of treated and untreated cells, respectively. All the experiments were performed in triplicate and the data are expressed as mean ± SE. The statistical differences among means have been analyzed by performing Student's *t*-test using GraphPad Software. The data represent significant values as ^*∗*^*p* < 0.05, ^*∗∗*^*p* < 0.01, and ^*∗∗∗*^*p* < 0.001, respectively.

## 3. Results

### 3.1. Isolation of Citral from *Cymbopogon citratus* Oil

The major active constituent citral was isolated and identified through various spectroscopic analyses, including electron ionized mass spectrometry (EI-MS) and nuclear magnetic resonance (NMR) spectroscopy. Citral was identified based on the following evidence: a colorless oil; UV (methanol): *λ*_max_ nm = 233; EI-MS (70 eV), *m*/*z* (% relative intensity): 152 [M]^+^ (6.4), 137 (3.8), 94 (12.5), 84 (24.6), 69 (100), 59 (3.40), 41 (87.3), ^1^H NMR (DMSO, 600 MHz): *δ* 1.62 (3H, s), 1.67 (3H, *d*, *J* = 1.2 Hz), 2.00 (2H, s), 2.18 (3H, m), 2.62 (2H, t), 5.20 (^1^H, m), 5.83 (^1^H, t), 9.96 (^1^H, d, *J* = 8.16 Hz). ^13^C NMR (CDCl_3_, 150 MHz): *δ* 192.4 d (C-1), 166.1 s (C-3), 134.4 s (C-7), 128.4 d (C-2), 123.5 d (C-6), 40.4 t (C-4), 26.9 t (C-5), 25.1 q (C-8), 18.1 q (C-9), 17.8 q (C-10) (Supplementary [Supplementary-material supplementary-material-1]).

### 3.2. Pharmacokinetic and Biological Activity Prediction of Neral and Geranial

**Figure 1:**
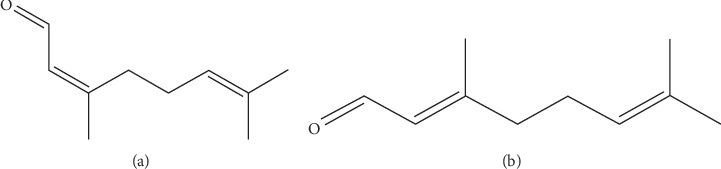
Structure of *cis-* and *trans*-citral. (a) Neral. (b) Geranial.

*Cis*-citral and *trans*-citral (Figure 1) molecules were further evaluated for their drug-like behavior and pharmacokinetic properties using SwissADME and PreADMET web servers based on relevant chemical descriptor. The calculated properties such as molecular weight, octanol/water partition coefficient, plasma protein binding (PPB) (<90%: weakly bound chemicals; >90%: strongly bound chemicals), human intestinal absorption (HIA) (0∼20% is poorly absorbed, 20∼70% is moderately absorbed, and 70∼100% is well absorbed), CYP2D6 inhibition (either inhibitor or noninhibitor), toxicity based on rodent carcinogenicity (+ is noncarcinogen and – is carcinogen), and blood-brain barrier (BBB) activity in CNS (>2.0 is high absorption, 2.0 ∼ 0.1 is middle absorption, and <0.1 is low absorption) were measured for citral molecule. In addition, Lipinski's rule of five was used for the determination of drug-likeness properties, such as molecular weight less than 500, fewer than 5 log *p* value octanol-water partition coefficient, fewer than 5 hydrogen bond donors, and fewer than 10 hydrogen bond acceptors. The detailed analyses of predicted ADMET values of neral and geranial with acceptable range are given in Table 2. All possible biological activities were predicted with probability of active (*P*_a_) and probability of inactive (*P*_i_) scores using PASS program. The predicted probability score ranges from 0 to 1 and *P*_a_ > 0.7 probability scores considered that our molecule has high chance to act as a biological function. The accuracy neral and geranial biological activity measured based on the *P*_a_ score and the biological activity related to the apoptosis induced cancer were listed in Table 3. Finally, drug-likeness, ADMET, and predicted biological activity results showed that neral and geranial molecules can act as potential drug-like molecule and considered for further experiments.

### 3.3. Molecular Interactions of Neral and Geranial

**Figure 2:**
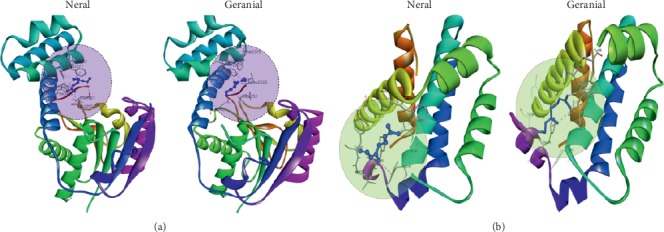
Molecular interaction studies of neral and geranial. (a) FASN. (b) Bcl-2. The amino acids involved in hydrogen bonding formation were represented in respective three-letter codes and the arrows indicate their hydrogen bond interaction. The three-dimensional protein structures and ligands were visualized in solid ribbon style followed by a rainbow color and ball and stick style (blue), respectively.

The crystal structure of FASN and Bcl-2 was used to conduct molecular docking simulation using AutoDock Vina. The known inhibitors of navitoclax (ABT-263) and orlistat structural coordinates were retrieved from Bcl-2 and FASN protein structures and used as control ligands for docking reproducibility, while checking the interaction results of the known inhibitor navitoclax (Bcl-2) and orlistat (FASN) yielded a binding affinity of −9.6 kcal/mol and −6.4 kcal/mol, respectively. Further, protein-ligand molecular interactions were confirmed by their binding affinity and interaction residues between the active site residues of proteins. Our result indicates that both neral and geranial have interacted with FASN (Figure 2(a)) with −6.3 Kcal/mol and −6.4 Kcal/mol, respectively. The neral and geranial interaction with Bcl-2 (Figure 2(b)) yielded a docking score that was found to be −5.2 Kcal/mol and −5.3 Kcal/mol, respectively (Table 4). Based on these results, we have taken our study to the next level to identify the role of citral isomers in prostate cancers.

### 3.4. Citral Inhibited Cell Viability, Proliferation, and Clonogenic Potential of Prostate Cancer (PC3 and PC-3M) Cells

**Figure 3:**
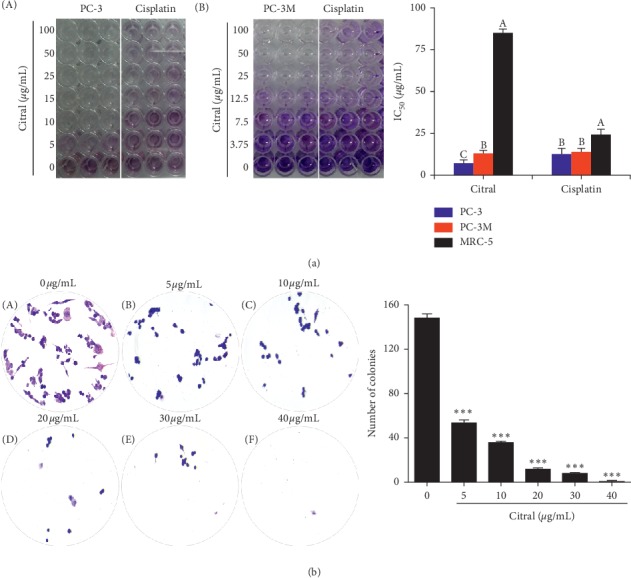
Citral treatment resulted in cell viability and decreased colony formation. (a) Inhibitory concentration (IC_50_) of citral of PC3 and PC3M prostate cancer cells was compared with positive control cisplatin. Cell toxicity of citral was compared with normal cell line MRC-5. (b) Citral inhibited the clonogenic formation of prostate cells in a dose-dependent manner. Colonies were stained with crystal violet staining and counted using ImageJ software. Each bar represents the mean ± SE of duplicate samples of three independent experiments (^*∗∗∗*^*p* < 0.001 using Student's *t*-test).

To determine the effect of citral on the proliferation of prostate cancer cells (PC3 and PC-3M), MTT assay was performed. We determined that the treatment of citral in a dose-dependent manner inhibited the cell viability of prostate cancer cells (Figure 3(a)). After 72 hours of incubation, IC_50_ of citral in PC3 and PC-3M cells was found to be 10 and 12.5 *μ*g/ml, respectively (Figure 3(a)). The citral showed strong inhibitory action in PC3 compared to PC-3M. Later, we performed colony formation assay to evaluate the clonogenic survival of prostate cancer cells after citral treatment. From the result, we found that citral can inhibit the colony formation of PC3 cells in a dose-dependent manner (Figure 3(b)). Results indicated that citral reduced the colony formation significantly compared to that of untreated cancer cells (Figure 3(b)). These results conclude that citral treatment to prostate cancer cells reduces the cell viability, proliferation, and clonogenic potential of cancer cells.

### 3.5. Citral Induced Altered Morphological Characteristics of Cancer Cells

**Figure 4:**
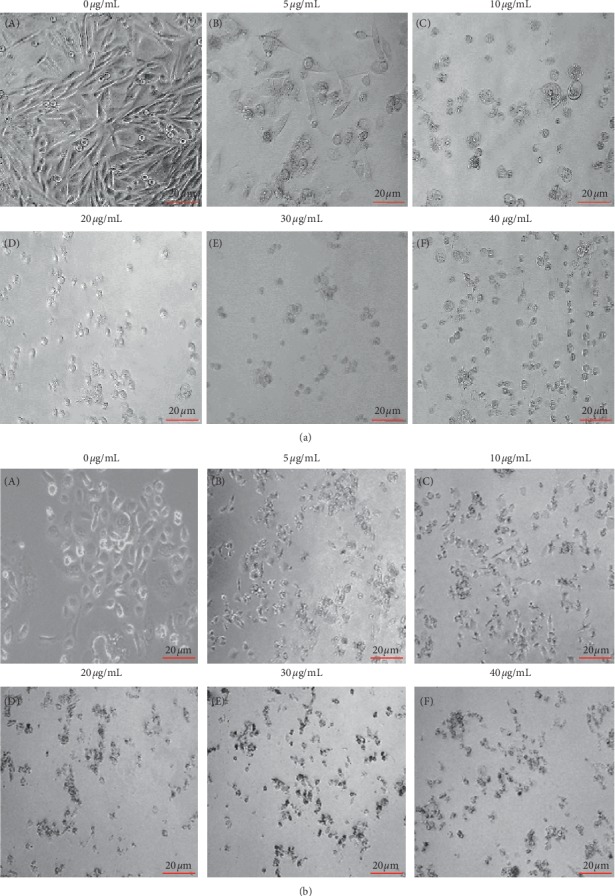
Morphological changes in prostate cancer cells. Morphological characteristics of prostate cancer cells (PC3M and PC3) were observed with a phase-contrast microscope (a) and (b). The inhibition of growth by treating different concentrations of citral was observed by showing distinct morphology. Images are representative of three independent replicates.

The morphological characteristics of prostate cancer cells were monitored after different concentrations of citral treatment. The results showed that both PC3 and PC3M cells upon 5 *μ*g/ml citral treatment exhibited gradual increase in morphological alteration and reached its maximum when cells were treated with 100 *μ*g/ml of citral. When compared to untreated cells which showed 90% confluent, treated cells exhibited distinct morphological pattern including shrunken and shape-less phenotype. In addition, the cell size and number were also significantly reduced which showed that citral resulted in the detachment of cells and caused alternation in morphology (Figures 4(a) and 4(b)).

### 3.6. Citral Inhibited Lipogenesis of Cancer Cells

**Figure 5:**
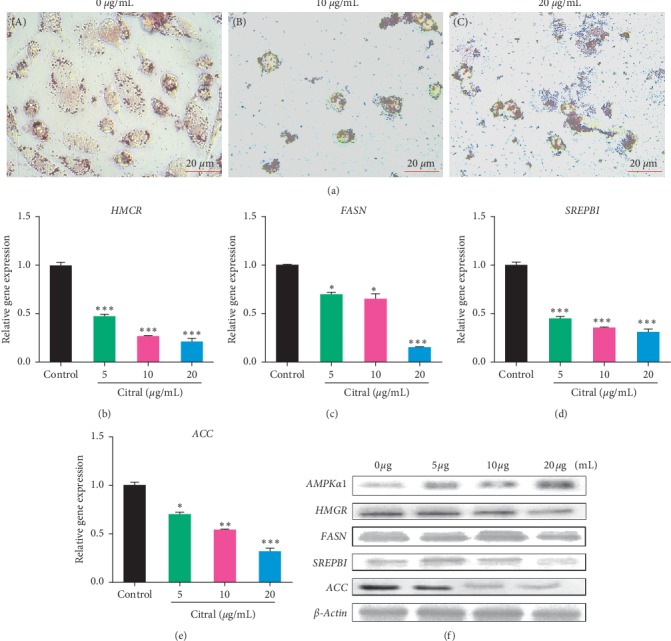
Citral suppressed lipogenesis of prostate cancer cells. (a) Oil Red O staining was used to identify the lipid droplets in prostate cancer cells (PC3) with (65.7 and 131.4 *µ*M) or without treatment. (b–e) The mRNA expression analysis of various transcripts involved in the lipogenesis pathway was quantified using qPCR analysis. The *β-*actin was used as an internal control. (f) The protein expression of AMPK-P, HMGR, FASN, SREBP1, and ACC was quantified using Western blotting. Each bar represents the mean ± standard error of triplicate samples from three independent experiments (*p*=0.05, using Student's *t*-test).

During cancer, an abnormal cell proliferation happens for which lipogenesis pathway plays a crucial role. To determine how the citral treatment interferes on lipid metabolism of cancer cells, we treated PC3 cells with two different concentrations of citral (10 and 20 *μ*g/ml) for 2 days, and ORO staining was carried out. The control cells clearly showed the accumulation of lipid droplets in an intact cell (Figure 5(a)-A). However, when the cells were treated with citral, the cells were significantly damaged and resulted in the expulsion of lipid droplets and thus inhibited the survival of cancer cell (Figure 5(a)-B and C). To further confirm the role of citral on the inhibition of lipogenesis, we have studied the genes and proteins involved in AMPK pathway. In Figure 5(f), the activation of AMPK protein expression resulted in the downregulation of AMPK pathway genes such as *SREBP1* (0.308-fold), *ACC* (0.32 fold), and *HMGR* (0.212-fold), respectively (Figures 5(b)–5(e)), and its downregulation is well correlated with decreased protein expression (Figure 5(f)) except *FASN* (0.15-fold) which showed very less protein expression at high concentration compared with other genes.

### 3.7. Citral Treatment Induced Apoptosis of Cancer Cells

**Figure 6:**
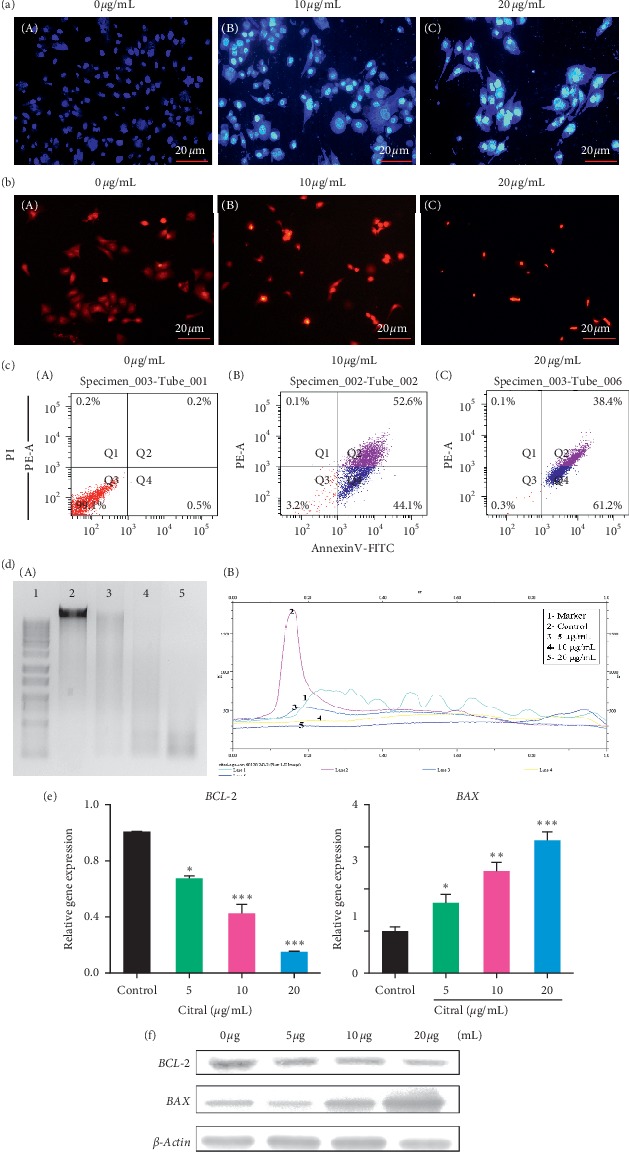
Citral induced apoptosis of prostate cancer cells. (a) Nuclear staining of prostate cancer cells with or without citral treatment using Hoechst dye. (b) Prostate cancer cells with or without treatment were stained with PI staining. The cells that remained intact does not allow cells to stain with the dye. However, the damage cells were stained with PI and indicated the apoptotic cells. (c) Flow cytometry analysis of cancer cells with or without treatment. Descriptive figures show the population of live cells (annexin V- PI-), early apoptotic (annexin V + PI-), late apoptotic cells (annexin V + PI+), and necrotic cells (annexin V-PI+). (d) DNA fragmentation analysis was carried out after the isolation of genomic DNA from prostate cancer cells with or without different concentrations of citral treatment. (e) The gene expression analysis of *Bcl-2* and *BAX* was carried out in cells with or without citral treatment. Housekeeping gene β-actin was used as an internal control. (f) The Western blotting analysis performed using antibodies indicated in Section 2. Each bar represents the mean ± standard error of triplicate samples from three independent experiments (*p*=0.05, using Student's *t*-test).

To determine citral treatment induced apoptosis, we used various apoptosis detection methods to identify whether citral induced cancer cell death was due to apoptosis. At first, we used Hoechst staining to confirm citral initiated apoptosis in cancer cells. Under nontreated condition, the cancer cell membrane remained intact such that the dye was unable to diffuse into the nuclei and hence no fluorescence was detected (Figure 6(a)-A). In contrast, cancer cells treated with different concentrations of citral caused cell membrane damage and thus the dye was able to penetrate the cell and thus stained the nuclei (Figure 6(a)-B and C). Secondly, we used PI staining to determine the cell viability and apoptosis of both treated and untreated cells. Control cells do not exhibit any fluorescence when stained with PI indicating that the cells remained intact (Figure 6(b)-A). Cells treated with 10 or 20 *μ*g/ml of citral reduced the cell viability and cell number indicating that citral treatment damaged cell membrane and thus resulted in red fluorescence (Figure 6(b)-B and C). Next, we performed annexin-V-FITC labeling to identify both early and late stage apoptosis on citral treatment. The untreated cells showed 99.1% of live cells with at least early and late apoptosis and necrosis (Figure 6(c)-A). However, the cells treated with 10 or 20 *μ*g/ml of citral exhibited the significant accumulation of late apoptotic cell (33.6% and 60.1%) and early apoptotic cell (53.5% and 44.9%), respectively (Figure 6(c)-B and C). Later, DNA fragmentation assay was performed for both treated and nontreated cancer cells. Control wells possessed intact DNA which is devoid of degradation. However, the cells treated with different concentrations of citral resulted in fragmentation of DNA in a dose-dependent manner (Figure 6(d)-A and B). Finally, we studied the genes and proteins that are regulated by citral treatment. The different concentrations of citral (5, 10 and 20 *μ*g/ml) were treated to PC-3 cells for 48 h and mRNA expression patterns of apoptosis related pathway genes such as *BAX* (Bcl-2-associated X protein) and *Bcl-2* were analyzed. In 5 *μ*g/ml treatment, the *Bcl-2* gene gradually decreased up to 0.67-fold and greatly decreased up to 0.15-fold with 20 *μ*g/ml of treatment (Figure 6(e). In contrast, the expression profile of *BAX* was also upregulated in a gradual manner (Figure 6(e)). To determine whether the alteration in gene pattern changes protein expression, we performed Western blot analysis. From the results, we conclude that treatment of citral has resulted in the induction of BAX and downregulation of Bcl-2 (Figure 6(f)).

## 4. Discussion

In the 1990s, prostate cancer was not considered as an important issue in Asia due to its low prevalence rate [1]. However, in recent years, a dramatic increase of prostate cancer has drained the attention of many researchers to actively participate in prostate cancer related researches. According to the National Health Insurance Corporation, the number of prostate cancer patients in outpatient clinics increased from 4843 men (2002) to 20498 men (2010), respectively. The current treatments for treating prostate cancer are limited because the patients poorly respond to the treatment and metastatic disease can gradually develop even after radical prostatectomy [27]. Therefore, identifying the drug target that kills the metastatic cells is extremely important to overcome this deadly disease. Complementary and alternatives medicines from traditional medicinal plants containing active bioactive principles aid to target the molecular pathways in cancer and thus prevent the progression of the disease with little or no side effects [28].

Citral is a mixture of two stereoisomeric monoterpene aldehydes such as the *cis*-citral (neral) and *trans*-citral (geranial). It is also found in several plants including Melissa and lemon. Lemongrass oil is used as the main ingredient in cosmetics, food, and perfumes and has a wide range of therapeutic properties including antibacterial, antifungal, analgesic, and antispasmodic properties and has an effect on the central nervous system not only on cancer. The therapeutic use of this oil was due to the presence of monoterpenes and myrcene. Few studies demonstrated the anticancer properties of citral in various cancer cell lines including breast (MDI-MB-231), small-cell lung cancer, colorectal cancer (HCT116 and HT29), and cervical cancer (HeLa and ME-180) [12, 29–31]. Although various studies in a preliminary level of citral have been studied against various cancer cell lines, the effect of *cis-* and *trans*-citral on aggressive prostate cancer (PC3) has not been elucidated so far. In our present study, we have isolated monoterpene (citral) from lemongrass and investigated the anticancer effects against prostate cancer cells. Our results conclude that citral treated to PC3 induced apoptosis via the lipogenesis pathway.

Studying cancer metabolism has become a new trend in the field of cancer research [32]. In general, cancer cell metabolism greatly varies from that of normal cells which involves abnormal activation of glycolysis and elevated lipid biosynthesis by modulating various genes and proteins [33]. Prostate cancers are highly dependent on altered lipid metabolism and enhanced cholesterol biosynthesis for its survival [34]. Understanding how lipid biosynthesis influences the proliferation of cancer cells will aid in attaining the knowledge of the link between cellular metabolism and inhibition of cancer cell growth, ultimately leading to the development of drugs in human cancer treatments. With the previous evidence, there is a significant motivation developed to investigate the changes in lipid metabolism of prostate cancer with or without citral isomers treatment. During the last decade, targeting the apoptosis pathway is considered a potential way to treat various diseases including cancers. For this purpose, we have isolated and identified the active principle from lemongrass oil. The constituent was determined to be citral which is a monoterpene that exhibited significant antiproliferative activity toward prostate cancer cell lines (PC-3 and PC3M).

To attain the knowledge of how citral isomers alter cancer cell metabolism and result in apoptosis, we used *in silico* studies to identify whether neral and geranial could be target drugs by showing strong biological activity, drug candidates, and essential role in cancer cell metabolism by undergoing apoptosis. At first, the effect of citral on biological activity was studied using the PASS program and predicted that citral showed strong biological activity in inhibiting lipogenesis and inducing apoptosis (Table 3). The bioavailability of the drug is essential for any drug to exhibit pharmacological property. Therefore, we studied ADMET to predict the drug likeliness of *cis*-citral and *trans*-citral. From Table 2, we identified that both *cis* and *trans* forms were well absorbed in the intestine (100%) and have a strong binding capacity (100%). For the protein-ligand interaction studies, we have selected the specific target proteins which are involved in lipogenesis and apoptosis pathway including FASN and Bcl-2 because prostate cancer survival depends on the lipogenesis pathway. Interaction studies demonstrated that *cis*-neral and *trans*-geranial could bind to the active site of each protein and thus left the way to study these proteins *in vitro*.

Several studies demonstrated that the upregulation of genes involved in lipogenesis and cholesterol synthesis is essential for the survival of a wide variety of tumor cells. Interestingly, there is no cytotoxicity of normal cells developed while inhibiting the expression of FASN and ACC. This denotes that normal cell growth relies on the fat from the dietary or culture medium and is less dependent on de novo fatty acid synthesis whereas the cancer cells purely addicted to de novo fatty acid synthesis. Fatty acid synthesis is associated with the activation of AMPK, a crucial enzyme that is required for modulating AMP/ATP ratio in the fatty acid synthesis [35]. A previous report suggests that the activation of AMPK through phosphorylation inhibits ACC, FASN, SREBP1, and other lipogenesis enzymes, which is the distinctive feature of most of the tumor cells [36, 37]. Studies have shown that the activators of AMPK such as 5-aminoimidazole-4-carboxamide riboside or the thiazolidinedione rosiglitazone could diminish the lipid synthesis including phospholipids and cholesterol and thus result in the inhibition of cell migration and proliferation of cancer cell. These findings suggest that AMPK is the right target to consider in the treatment of cancer. Activated AMPK directly phosphorylates (AMP and ADP) a number of substrates to acutely impact the metabolism and growth, as well as phosphorylating a number of transcriptional regulators that mediate long-term metabolic reprogramming [38]. When elevated AMP is bound to the *γ* subunit, the inhibitory domain of the *α*1 subunit is released from the kinase domain. This results in an active conformation of AMPK, which allows the upstream kinases, such as LKB1, to phosphorylate Thr172 on AMPK*α* [39]. Our present study showed that the treatment of citral activated AMPK phosphorylation by the activation of gene and protein levels and thus inhibited the enzymes involved in fatty acid and cholesterol biosynthesis such as SREBP1 and HMGR. Additionally, the activation of AMPK also resulted in the activation of BAX and downregulated the Bcl-2 and thus resulted in apoptosis. The apoptosis induction by citral was also inconsistent with the results of Hoechst, PI staining which caused fluorescence in dead and damaged nuclei, FACS which identified early and late apoptotic cells, and DNA fragmentation which visualized degradation of DNA (Figures 6(a)–6(f)).

## 5. Conclusion

**Figure 7:**
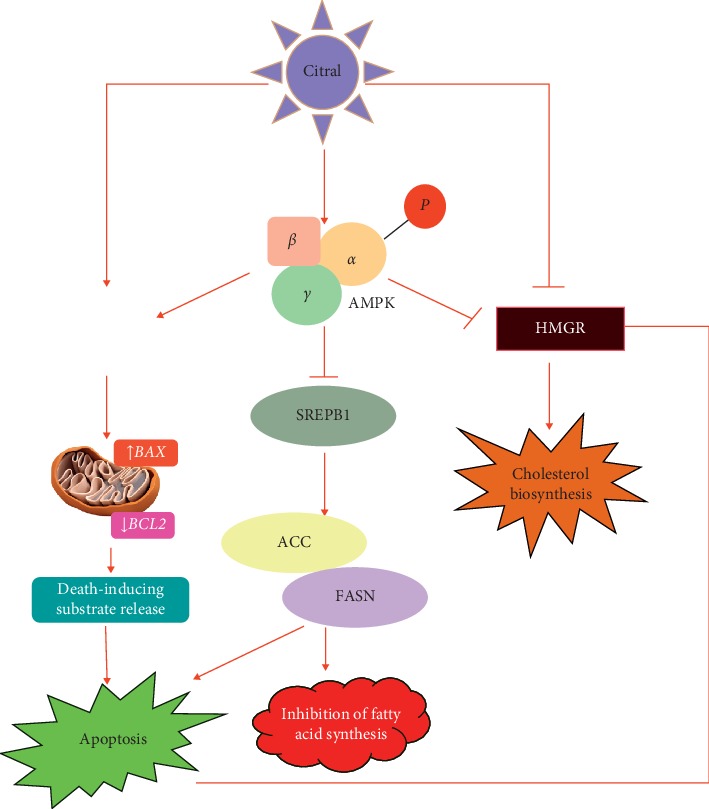
Tumor suppressing pathway of citral on prostate cancer cells. Upon citral treatment, the prostate cancer cell activates phosphorylation of AMPK and inhibits fatty acid synthesis and cholesterol pathway genes such as *SREBP1*, *ACC*, *FASN*, and *HMGR* and that in turn increases the expression of BAX, downregulates Bcl-2 expression, and promotes apoptosis.

We have isolated active ingredient citral from lemongrass oil and studied its anticancer properties against human prostate cancer cells. Typically, our results indicate that citral from lemongrass induces apoptosis driving lipogenesis pathway in both *in silico* and *in vitro* analyses. The citral suppressed colony formation, inhibited lipogenesis, and induced cell death through apoptosis. Induction of AMPK and downregulation of crucial genes involved in lipogenesis resulted in apoptosis exhibiting antiproliferative effects of citral (Figure 7). It is also noteworthy to suggest citral as a promising candidate as it absorbs well in the intestine and can exhibit its anticancer effects on prostate cancer cells. It will be interesting to investigate the *in vivo* action of citral against prostate cancer for the therapeutic use of citral as an anticancer drug.

## Figures and Tables

**Table 1:** Primers listed in the present study.

S.no.	Genes	Forward primer (5′-3′)	Reverse primer (5′-3′)
1	*HMGR*	CTTGTTCATGCTCACAGTCG	ACCAGCATAGGTTCACGTCTA
2	*FASN*	AACGGCAACCTGGTAGTGAG	GTGTCCATGAAGCTCACCCA
3	*SREBP1*	GATGCGGAGAAGCTGCCTAT	GCTGTGTTGCAGAAAGCGAA
4	*ACC*	TGGTTCTTGGGTTGTGATCGA	TCGGTCAGCGTACATCTCCAT
5	*BAX*	TTCTGACGGCAACTTCAACTG	GTTCTGATCAGTTCCGGCA
6	*Bcl-2*	AGCACTCCCGCCACAAAGA	GAGGCAAGCATAAGACTGG
7	*β-actin*	CATCACTATCGGCAATGAGC	GACAGCACTGTGTTGGCATA

**Table 2:** ADMET characteristic of *cis*-citral and *trans*-citral.

Molecule	Molecular weight	Log *P*_o/w_	Plasma protein binding (%)	Human intestine absorption (%)	CYP2D6	Toxicity (R)	Blood-brain barrier	Lipinski's violations
*cis*-citral (neral)	154.25	2.74	100	100	Noninhibitor	−	6.7413	Nil
*trans*-citral (geranial)	152.23	2.71	100	100	Noninhibitor	+	2.00829	Nil

Log *P*_o/w_: octanol/water partition coefficient; PPB: plasma protein binding (<90% is weakly bound chemicals and >90% is strongly bound chemicals); HIA: human intestinal absorption (0∼20% is poorly absorbed, 20∼70% is moderately absorbed, and 70∼100% is well absorbed); toxicity: rodent carcinogenicity (R: Rat, + is noncarcinogen and–is carcinogen); BBB: blood-brain barrier activity in CNS (>2.0 is high absorption, 2.0 ∼ 0.1 is middle absorption, and <0.1 is low absorption).

**Table 3:** Prediction of activity spectra for substances (PASS).

S. no.	Predicted biological activity	*P *_a_	*P *_i_
1	Apoptosis agonist	0.826	0.006
2	Fatty-acyl-CoA synthase inhibitor	0.784	0.005
3	TP53 expression enhancer	0.747	0.018
4	Farnesyltransferase inhibitor	0.6559	0.002
5	Chemoprotective	0.625	0.004
6	MMP9 expression inhibitor	0.626	0.013
7	HMOX1 expression enhancer	0.616	0.017
8	Transcription factor stimulant	0.590	0.010
9	TNF expression inhibitor	0.590	0.014
10	Transcription factor NF kappa B stimulant	0.590	0.010
11	Immunostimulant	0.608	0.022
12	Lipid metabolism regulator	0.594	0.022
13	Caspase 3 stimulant	0.588	0.018
14	Anticarcinogenic	0.539	0.016
15	HMG CoA synthase inhibitor	0.382	0.004
16	Prostate cancer treatment	0.401	0.022
17	Antioxidant	0.385	0.013

**Table 4:** Molecular interaction analysis of neral and geranial against each target protein.

Proteins	Molecules	Docking energy (Kcal/mol)	Interaction residues
FASN	Neral	−6.3	ILE2250, LEU2251, PHE2323, PHE2370, PHE2371, ALA2419, PHE2423
	Geranial	−6.4	LEU2222, GLU2251, PHE2370, PHE2371, PHE2375

Bcl-2	Neral	−5.2	ALA97, ASP100, PHE101, TRP141, GLY142, VAL145, TYR199, PHE195, LEU198
	Geranial	−5.3	ALA97, PHE101, TYR105, TRP141, GLY142, VAL145, PHE195, LEU198, TYR199

## Data Availability

No data were used to support this study.

## Abbreviations

MTT: 3-(4,5-dimethylthiazol-2-yl)-2,5-diphenyl tetrazolium bromide
ADMET: Absorption, distribution, metabolism, excretion and toxicity
SREBP1: Sterol regulatory element-binding protein
FASN: Fatty acid synthase
BCL-2: B-cell lymphoma 2
BAX: Bcl-2-associated X protein
FBS: Fetal bovine serum
TLC: Thin-layer chromatography
RPMI: Roswell Park Memorial Institute
DMEM: Dulbecco's Modified Eagle's Medium
FBS: Fetal bovine serum
EDTA: Ethylenediaminetetraacetic acid
qRT-PCR: Quantitative real-time PCR
PPB: Protein binding ability
HIA: Human intestinal absorption
PASS: Prediction of activity spectra for substances
HRP: Horseradish peroxidase
CL: Confidence limits
IC_50_: Inhibition concentration
PS: Phosphatidylserine.

## Supplementary Materials

Supplementary Materials Supplementary Figure S1: NMR spectrum of citral. (A) ^1^H NMR. (B) 13 832 C NMR spectra.
