# Supplementary figures and images for: Citral Induced Apoptosis through Modulation of Key Genes Involved in Fatty Acid Biosynthesis in Human Prostate Cancer Cells: In Silico and In Vitro Study
Source: Biomed Res Int. 2020 Mar 18;2020:6040727. doi: 10.1155/2020/6040727 (PMC7103989; doi:10.1155/2020/6040727)

947

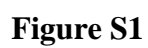

948

949

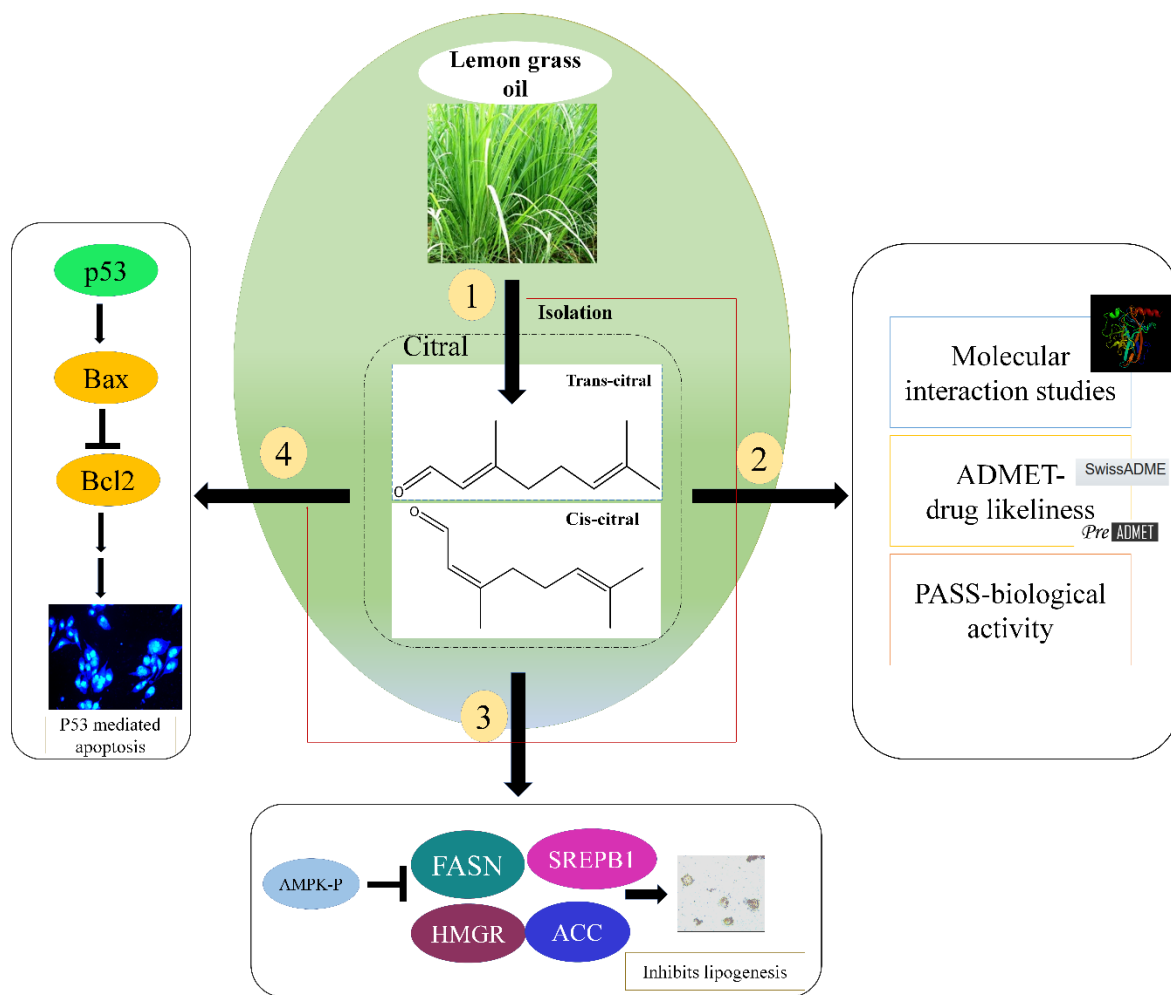

950

951

## Graphical abstract

Supplement: Supplementary Materials — Supplementary Figure S1: NMR spectrum of citral. (A) 1H NMR. (B) 13 832 C NMR spectra. [file 6040727.f1.pdf]
